# Preparation of DNC Solid Dispersion by a Mechanochemical Method with Glycyrrhizic Acid and Polyvinylpyrrolidone to Enhance Bioavailability and Activity

**DOI:** 10.3390/polym14102037

**Published:** 2022-05-16

**Authors:** Min Lu, Wei Wei, Wenhao Xu, Nikolay E. Polyakov, Alexandr V. Dushkin, Weike Su

**Affiliations:** 1 National Engineering Research Center for Process Development of Active Pharmaceutical Ingredients, Collaborative Innovation Center of Yangtze River Delta Region Green Pharmaceuticals, Zhejiang University of Technology, Hangzhou 310000, China; yaolilumin@163.com (M.L.); 13619587845@163.com (W.W.); xuwenhao@zjut.edu.cn (W.X.); avd@ngs.ru (A.V.D.); 2 Institute of Chemical Kinetics and Combustion, 630090 Novosibirsk, Russia; polyakov@kinetics.nsc.ru; 3 Institute of Solid State Chemistry and Mechanochemistry, 630090 Novosibirsk, Russia; 4 Key Laboratory for Green Pharmaceutical Technologies and Related Equipment of Ministry of Education, College of Pharmaceutical Sciences, Zhejiang University of Technology, Hangzhou 310000, China

**Keywords:** nicarbazin, DNC, glycyrrhizic acid, PVP, micelles, coccidiosis

## Abstract

To exploit aqueous-soluble formulation and improve the anticoccidial activity of 4,4′-dinitrocarbanilide (DNC, active component of nicarbazin), this paper prepared DNC/GA/PVP K30 solid dispersion (SD) with glycyrrhizic acid (GA) and polyvinylpyrrolidone (PVP) K30 by a mechanical ball milling method without using any organic solvent. Fourier transform infrared spectroscopy, X-ray diffraction, differential scanning calorimetry, and scanning electron microscopy were used for the solid state characterization. High performance liquid chromatography, critical micelle concentration, particle characterization, and transmission electron microscopy were used to evaluate the behavior in aqueous solution. In addition, the oral bioavailability, tissue distribution, and anticoccidial activity of DNC/GA/PVP K30 SD were investigated as well. Compared with free drug, the novel formulation not only improved the solubility and dissolution rate of DNC, but also inhibited the fecal output of oocysts and enhanced the therapeutic effect of coccidiosis. According to the experiment results, the DNC/GA/PVP K30 SD increased 4.64-fold in oral bioavailability and dramatically enhanced the concentration in liver which provided a basis for further research in schistosomiasis. In summary, our findings suggested that DNC/GA/PVP K30 SD may have promising applications in the treatment of coccidiosis.

## 1. Introduction

Chicken coccidiosis is one of the infectious parasite severe diseases and causes increasing morbidity and mortality every year in the poultry industry throughout the world, hampering the productivity and economic development [1]. As a leading parasite, *E. tenella* is the most pathogenic and fatal for seven species of coccidian parasite, which live inside the intestines’ tract of chicken, cause malabsorption of nutrients, enteritis, and fluid loss destruction, resulting in extensive hemorrhage and death [2]. These coccidian parasites are highly widespread and can persist for long periods in the environment, such as feces and litter, causing many chickens to become infected with more than 50% mortality rate [3,4], and the cost of coccidiosis in chickens is estimated to $3 billion USD per annum [1]. Therefore, control of the coccidiosis in poultry is essential for food security and economic development.

Nicarbazin (NIC) is a broad-spectrum anticoccidial agent with high security and low resistance, the equimolar complex of 4,4′-dinitrocarbanilide (DNC) with 2-hydroxy-4,6-dimethylpyrimidine (HDP), which has been synthesized and utilized since the 1950s [5]. Previous contribution has reported that NIC [6] inhibited the second generation schizont of *E. tenella*, which was essential for killing and restraining coccidia growth; therefore, NIC has been proposed as a promising preventing strategy for the therapy of coccidiosis. The active ingredient of NIC is DNC (a highly insoluble drug), and the other part HDP had no coccidioidal activity [7], hydrogen bonding between them greatly improving the anticoccidial effect compared to that of the administration of DNC alone [8]. However, the further application of DNC is restricted due to its poor solubility in aqueous, and improving the water solubility may enhance the drug efficacy. Formation of Solid dispersion (SD) has been widely used to increase aqueous solubility of hydrophobic drugs, which provided an important route to improve dissolution rate and bioavailability and reduce toxicity. Various manufacture approaches have been employed to produce solid dispersion, such as solvent evaporation, melting, etc. [9]. However, solvent evaporation will leave the product with residual organic solvent and high temperature of the melting process may lead to decomposition or degradation of the drug [10]. Recently, a novel strategy, mechanochemistry preparing SD widely applied as drug solubilization, has the remarkable characteristics of changing particle structure and enhancing the activity of drug by simple operation with high efficiency and less solvent residual [11]. Researchers prepared curcumin SD using the mechanochemical method; the bioavailability of curcumin increased 10 times, the corresponding water solubility has also been greatly improved; meanwhile, the SD had strong lipid-lowering ability and anticancer capacity compared with curcumin [12,13]. Xu et al. conducted a mechanochemical ball milling technique to achieve astaxanthin solid dispersion [14] and 5-amino salicylic acid pH-sensitive hydrogel [15] with higher aqueous solubility than pure drugs. Therefore, mechanical ball milling will be a great choice to enhance the water solubility of drugs under the conditions of being environmentally friendly and having high-performance productivity.

Glycyrrhizic acid (GA) is a kind of naturally occurring sweetener, as a triterpenoid glycoside had been extracted from glycurrhiza root with antiviral, anti-inflammatory, anticancer, and hepatoprotective activities et al. [16]. In a drug delivery system, GA has strong complexation ability for drugs and self-association in aqueous solution as an amphiphilic molecule could enhance the solubility and permeability of hydrophobic compounds, rendering the drug dispersed in aqueous solutions to form a stable and homogenous solution [17]. Yang et al. [18] had prepared Paclitaxel-loaded glycyrrhizic acid micelles to increase the bioavailability of paclitaxel. Zheng et al. reported a method to prepare inositol hexanicotinate SD with GA and arabic gum by mechanical ball milling to enhance the solubility and bioavailability. Polyvinylpyrrolidone (PVP) was obtained by radical polymerization of the monomer, N-vinyl-pyrrolidone, and proved to be a safe water-soluble polymer with a variety of molecular weight and viscosity [19,20]. The degree of polymerization and molecular weight of PVP determines its viscosity, which is represented by the K value. A great number of reports claimed that the application of PVP could enhance dissolution rate and bioavailability of drug, such as phenytoin, sulfathiazole, hydrocortisone, disulfiram, etc. [21]. Frizon et al. [22] prepared loratadine SD with PVP K30 to enhance its dissolution rate. Previous reports pointed out that PVP and tannic acid (TA) combined stabilization through hydrogen-bonding interactions between carbonyl groups (PVP) and hydroxyl groups (TA) [23]. However, whether glycyrrhizic acid, an excipient containing hydroxyl groups just like TA, is combined with PVP to change the physicochemical properties of drugs by mechanical ball milling has not been reported, which is a significant study for verifying this point.

In this study, DNC (the active compound of nicarbazin) was used as the model drug, using mechanical ball milling to prepare DNC SD with GA and PVP K30, then characterizing the solid state and evaluating the behavior in aqueous solution. Furthermore, the permeability and tissue distribution studies of DNC SD were conducted. The anticoccidial activity of DNC SD was investigated in vivo as well. Our finding indicated that DNC SD could self-assemble to form micelles when dissolved in water, meanwhile showing the improvement of aqueous solubility, dissolution rate, and oral bioavailability, which may have potential application in coccidiosis therapy.

## 2. Materials and Methods

### 2.1. Materials

**Figure 1:**
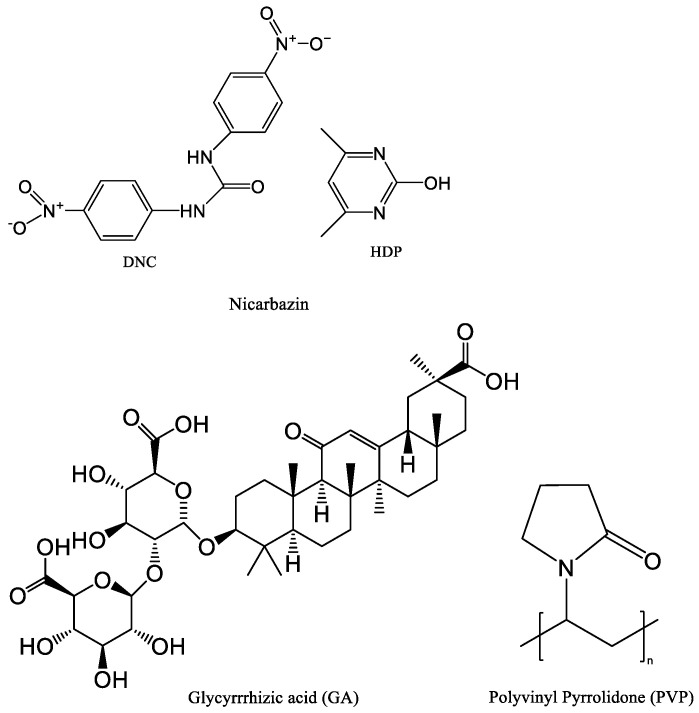
Structure of NIC, GA, and PVP.

Nicarbazin (NIC) was purchased from Wuhan Changcheng Chemical Technology Development Co., Ltd. (Wuhan, China, purity: 98%). Glycyrrhizic acid (GA) was purchased from Shaanxi Pioneer Biotechnology Co., Ltd. (Xi’an, China, purity ≥ 98%). PVP K30 was acquired from Meryer Co., Ltd. (Shanghai, China, purity: 100%). All other chemicals used were analytical grade. Figure 1 shows the chemical structures of NIC, GA, and PVP.

### 2.2. Preparation of the DNC Solid Dispersions (DNC SDs)

DNC solid dispersions were prepared by a planetary ball mill (PM 400, Retsch, Haan, Germany). Briefly, 0.44 g DNC and 1.76 g GA (mass ratio 1/4), or 0.37 g DNC, 1.46 g GA, and 0.37 g PVP K30 (mass ratio 1/4/1) were added to a 50 mL ball mill tank with 82 g steel balls (diameter 9 mm) with rotation speed 300 rpm, 30 min, were called DNC/GA SD and DNC/GA/PVP K30 SD, respectively. Physical mixing (PM): the ingredients were mixed (with same SD mass ratio) and called DNC/GA PM and DNC/GA/PVP K30 PM.

### 2.3. Analysis of DNC by HPLC

The amount of DNC was determined with a high-performance liquid chromatography (HPLC) system (Agilent 1200, Santa Clara, CA, USA) with a diode-array detector. Chromatography was performed on an Agilent C18 column (3.0 × 250 mm, 5 µm) at 25 °C. The mobile phase consisted of acetonitrile-water (60:40, *v*/*v*); the flow rate was 0.8 mL/min. The injection volume was 10 µL and the detection wavelength was 350 nm.

### 2.4. Content Test for DNC in Solid Dispersion

To determine the content of DNC in SDs after ball milling, a certain weight of DNC SDs samples completely dissolved in 10 mL of dimethyl sulfoxide (DMSO) and was then analyzed by HPLC.

### 2.5. Powder X-ray Diffraction (PXRD)

An X-ray diffractometer (DNC/GA, Bruker D2 PHASER, Karlsruhe, Germany; DNC/GA/PVP K30, D/max-Ultima IV, Rigaku, Japan) was used to collect the wide-angle XRD profiles. The samples were exposed to CuKa radiation under 30 kV and 10 mA. The 2-theta of a wide angle XRD was in the range of 3°–40° with a speed of 4°/min, with a 0.02° step size.

### 2.6. Differential Scanning Calorimetry (DSC)

Thermal analysis (TA) of the accurately weighted amounts samples (5 mg) were carried out by DSC with the TA Instruments (SERIES 2000, Mettler-Toledo, Columbus, OH, USA) in Atmosphere. Samples were heated from 40 °C to 350 °C (heating rate: 10 °C/min). 

### 2.7. Fourier Transform Infrared (FT-IR)

To measure the functional groups spectra of the samples used, a Nicolet iS50 Fourier spectrophotometer (Thermo Fisher Technology Co., Ltd., Waltham, MA, USA), in the range of 400 to 4000 cm^−1^ and the resolution was 2 cm^−1^. All samples were dried and prepared as thin tablets with KBr.

### 2.8. Nuclear Magnetic Resonance (NMR) Relaxation Study 

The NMR spectrum of DNC SDs was recorded on a Bruker NMR spectrometer, and samples were completely dissolved in D_2_O solution, and the spin–spin relaxation time (T_2_) was determined by the standard sequence Carr–Purcell–Meiboom–Gill (CPMG) of the Avance version of the Bruker pulse sequence library.

### 2.9. Scanning Electron Microscopy (SEM)

A Zeiss Gemini 500 field SEM (Carl Zeiss AG, Germany) was used to acquire the electronic images of samples. Before observation, the coating of samples with gold was performed.

### 2.10. Particle Size Distribution and Zeta Potential

Dissolving 1 mg sample in 1 mL pure water, the particle size, polydispersity index (PDI), and zeta potential of samples were measured by dynamic light scattering (DLS, Nano ZS90, Malvern Instruments, Malvern, UK).

### 2.11. Drug Encapsulation Efficiency (EE) and Loading Capacity (LC) Determination

The accurately weighed DNC SDs were dissolved in water, centrifuged, and then the supernatant was analyzed by HPLC. The EE and LC of DNC SDs were calculated according to Equations (1) and (2):

EE (%) = (weight of drug in nanoparticle)/(total weight of drug) × 100%(1)

LC (%) = (weight of drug in micelles)/(total weight of micelles) × 100% (2)

### 2.12. Solubility Determination

Excess DNC SD samples were added into a 20 mL Erlenmeyer flask with 10 mL distilled water, shaken (200 r) at 37 °C for 12 h. Then, the samples solution was filtered and analyzed by HPLC. 

### 2.13. Transmission Electron Microscope (TEM)

DNC micelles were placed on a copper grid covered with nitrocellulose. TEM (HT7700, Hitachi Co., Ltd., Tokyo, Japan) was used to evaluate the morphology of DNC micelles in water.

### 2.14. Determination of Critical Micelle Concentration (CMC)

Nile red (NR) was used as the fluorescent probe to determine the CMC of micelles. First, 0.4 mg NR was added to a 10 mL acetone solution; then, 30 μL NR solution were added to 10 brown vials and evaporated, with each vial added in a different concentration gradient of DNC micelles solution, avoiding light shaking for 24 h. The samples solution detected the fluorescence intensity at the wavelength of 620 nm and excited at 579 nm.

### 2.15. Dissolution Determination

Dissolution tests of DNC SDs samples were performed using a dissolution tester (RC-6, Tianjin Jingtuo Instrument Technology Co., Ltd., Tianjin, China) at the paddle rotation speed of 100 rpm in 900 mL of distilled water at 37 ± 0.5 °C. Samples equivalent to 10 mg DNC were added to 900 mL of dissolution medium. At 5, 10, 15, 30, 45, 60, 90, and 120 min, 1 mL dissolution medium was withdrawn and an equal volume of distilled water was added. The collected samples were filtered and then analyzed by HPLC.

### 2.16. Parallel Artificial Membrane Permeability Assay (PAMPA)

The PAMPA experiment was used to predict the passive intestinal absorption of DNC SDs with transwell inserts (polycarbonate membrane, 6.5 mm, 0.4 μm pore size, Corning Incorporated). For the preparation of artificial membrane, each donor plate hole had 60 μL 5% added (2% DOPC in hexadecane) hexadecane/hexane solution (*v*/*v*), which evaporated completely. Then, each acceptor plate had 1 mL distilled water added, and the samples were added to the donor plate hole, shaken (25 °C, 200 rpm), taken out at 0.5, 1, 1.5, 2, 2.5, 3, 3.5, 4, 4.5, and 5 h, and evaluated by HPLC [13]. 

### 2.17. Animal Experiments

#### 2.17.1. Bioavailability Study

Healthy male Sprague–Dawley (SD) rats with the weight of 200 ± 20 g were provided by the Zhejiang Academy of Medical Science. All the experiments involving SD rats were performed in accordance with protocols approved by the Ethics Committee of Zhejiang University of Technology (Certificate number: 201907). 

SD rats were fasted 12 h before operation and randomly divided into 5 groups (n = 6 per group) as follows: DNC, NIC, NIC commercial product (NIC CP), DNC/GA, and DNC/GA/PVP K30 group with oral administration 0.5% CMC-Na suspension of DNC (90 mg/kg) equivalent weight. Blood was collected from the eyelids at different time points: 0.5, 1, 2, 4, 8, 12, 24, 48, and 72 h, then centrifuged (4 °C, 10,000 rpm for 10 min) with supernatant collected, and the plasma samples were stored at −80 °C and applied for determination of the DNC content. In addition, 1 mL methanol was added to a 100 μL plasma sample, and the solution was vortexed and centrifuged at 10,000 rpm for 10 min. The supernatant was collected and the content of DNC in the plasma was determined by ultra-high performance liquid chromatography-tandem mass spectrometry (UPLC-MS, ACQUITY H-Class/Xevo TQS, Waters Corporation, Milford, MA, USA). The UPLC system was as follows: chromatography was performed on a C18 column (2.1 × 50 mm, 1.7 μm, Acquity UPLC), column temperature was 30 °C, with methanol–water (90:10, *v*/*v*) as the mobile phase, and the flow rate was 0.2 mL/min. MS analysis: electrospray ionization (ESI) with positive mode; capillary voltage, 3 kV; source temperature, 150 °C desolvation temperature, 350 °C; gas flow rate, 650 L/H.

#### 2.17.2. Tissue Distribution Experiment 

Thirty SD rats were fasted 12 h and randomly divided into 5 groups (n = 6 per group) as follows: DNC, NIC, and NIC commercial product, DNC/GA and DNC/GA/PVP K30 group with oral administration 0.5% CMC-Na suspension of DNC (90 mg/kg) equivalent weight for 7 days in a row. After the experiment was over, all animals were euthanized using CO_2_ following IACUC guidelines. The heart, liver, spleen, lungs, and kidneys were dissected and washed clean by normal saline (NS), wiped with filter paper. In addition, 0.2 g tissues were weighed and mixed with 1 mL NS and homogenized using a tissue grinder to obtain tissue homogenate. Furthermore, 0.5 mL tissue homogenate was added to 1 mL methanol and then homogenized and centrifuged at 4 °C at 10,000 rpm for 10 min; the supernatant was used to determine the content of DNC in the organs by UPLC-MS.

#### 2.17.3. Anti-Occidial Activity

Twelve-day-old male broilers were weighed and assigned to 5 groups (n = 15 per group): negative control group (NC) with normal diet, positive control group (PC) with normal diet, DNC group (90 mg DNC /kg diet), NIC commercial product (NIC CP) group (125 mg NIC/kg diet) and DNC/GA/PVP K30 groups (540 mg DNC/GA/PVP K30 SD/L water). On day 1, each chick was orally challenged with 50,000 *Eimeia tenella* live oocysts except the NC group. The NC group was gavaged with distilled water at an equal volume. The clinical symptoms were recorded such as bloody stools, anorexia, huddling together, and disheveled feathers. The feces from days 7 to 9 were collected, and the amount of oocyst per gram (OPG) was measured according to the McMaster technique [24]. On the 9th day of the test, the chicks were weighed and sacrificed, the cecum dissected and scored, and the anticoccidial index (ACI) was calculated according to Equation (3) as well. 

Survival rate (*100%) is the ratio of the number of surviving chickens to the initial number of chickens. The relative weight gain rate is the ratio of the average weight gain of the infected group to the average weight gain of the NC group. The lesions score included hemorrhages, thickening of the cecum wall and mucoid discharge, ranging from 0 to 4. Oocysts percent gram converts to oocysts value according to Table 1. The ACI value [24,25] was ordered in different grades: ACI < 120 (inactive), 120 < ACI < 140 (mild), 140 < ACI < 160 (moderate), and ACI > 180 (excellent).

ACI = survival rate + relative weight gain rate (RWGR) − (lesions score + oocysts value)(3)

### 2.18. Stability Test of DNC SDs 

The same net weight of DNC/GA/PVP K30 SD was packed in aluminum foil bags, stored in a drug stability test chamber at 40 °C, 75% humidity, and the drug content, particle size, zeta potential, and X-ray diffraction were measured at 1, 2, and 3 months. 

### 2.19. Statistical Analysis 

PKSolver (China Pharmaceutical University, Nanjing, China) was used to calculate the parameters of bioavailability experiments. The data statistical analyses were performed with IBM SPSS Statistics 26. After one-way analysis of variance (ANOVA), a two-tailed Student’s *t*-test was used. Results were reported as the means ± S.E.M., and it is significantly different when the value of *p* < 0.05.

## 3. Results and Discussion

### 3.1. DSC

**Figure 2:**
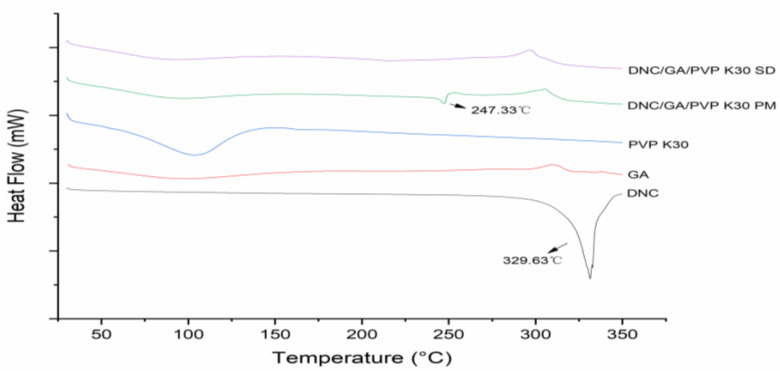
DSC curves of DNC, GA, PVP K30, DNC/GA/PVP K30 PM, and DNC/GA/PVP K30 SD.

DSC thermograms of all solid samples were shown in Figure 2. The endothermic peak of pure DNC exhibited approximately 329.63 °C with a ΔH value of 708.13 J·g^−1^. After adding GA and PVP K30, the endothermic peak of crystalline DNC still existed in the physical mixture because of the dilution effect. After ball milling, the intensity of DNC peak completely disappeared and ΔH values dropped to nearly 0 J·g^−1^, certifying that DNC had converted to a partly amorphous state by the mechanical ball milling.

### 3.2. PXRD

**Figure 3:**
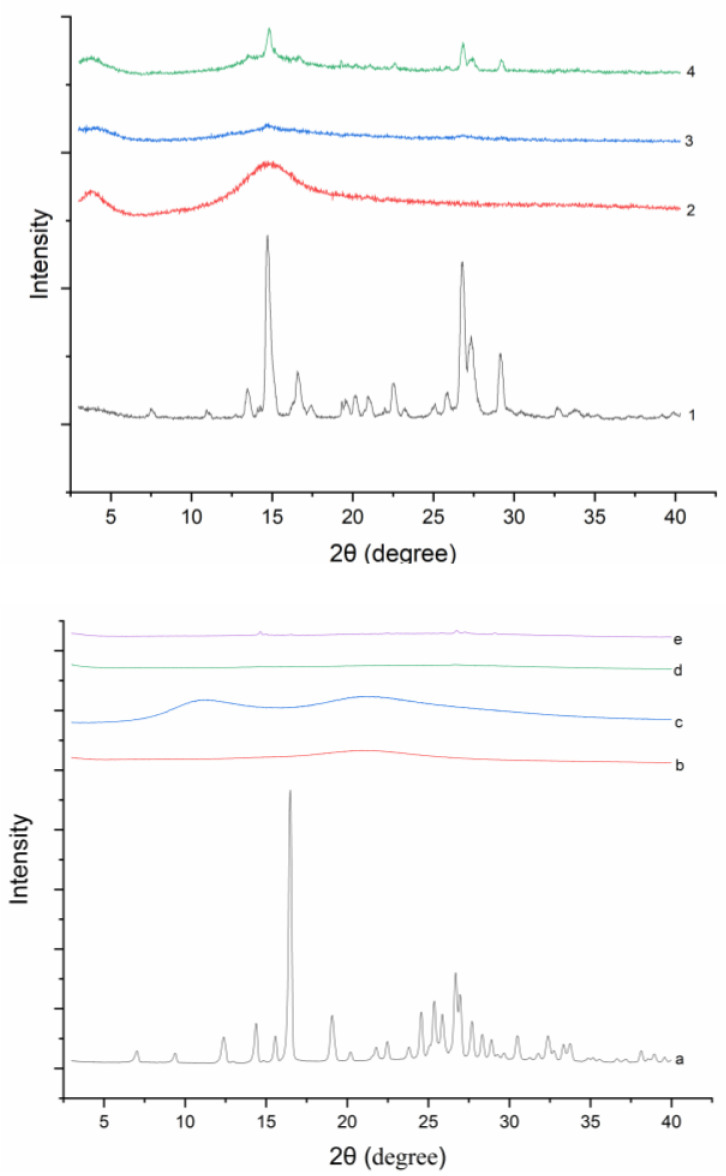
PXRD curves of (1, a) DNC, (2, b) GA, (3) DNC/GA SD, (4) DNC/GA PM, (c) PVP K30, (d) DNC/GA/PVP K30 SD, (e) DNC/GA/PVP K30 PM.

Powder X-ray diffractograms of all solid samples were shown in Figure 3. DNC has characteristic peaks at 2θ angles of 7.06, 9.42, 12.4, 16.5, 19.1, 24.6, 25.9, etc. DNC/GA PM and DNC/GA/PVP K30 PM showed several characteristic peaks as well, but the crystalline peaks of DNC SDs were drastically decreased or even disappeared. Both diffractograms and thermograms of DNC SDs’ results illustrated the conversion of the DNC to an amorphous dispersion by mechanical ball milling. Previous studies had showed that the amorphous state of drug could enhance the water solubility and dissolution [21]. Zhang et al. prepared amorphous camptothecin solid dispersion to increase the solubility of camptothecin by 178-fold and the cumulative release amount reached 46% [13].

### 3.3. FT-IR

**Figure 4:**
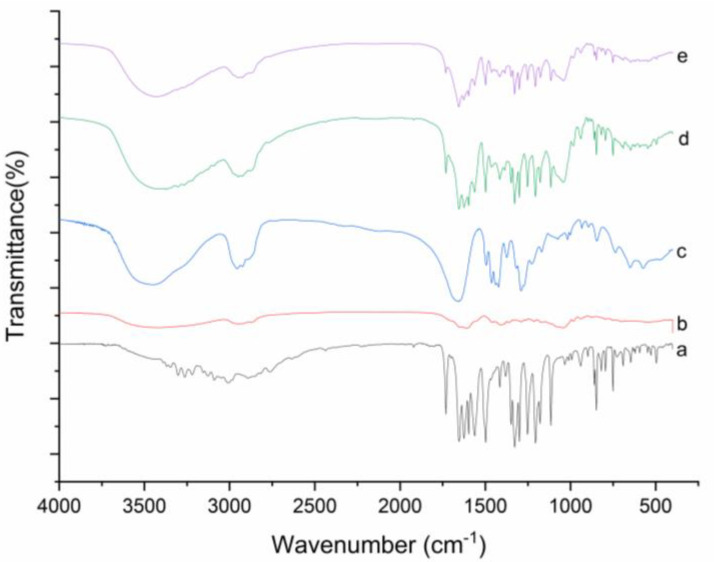
FT-IR spectra of (**a**) DNC, (**b**) GA, (**c**) PVP K30, (**d**) DNC/GA/PVP K30 SD, and (**e**) DNC/GA/PVP K30 SD.

FT-IR spectroscopy was used to estimate the possible by-products in the ball milled DNC SDs, with the infrared spectrum of pure DNC, GA, PVP K30, and DNC PMs above, Figure 4. Pure DNC has strong tensile vibration peaks at 1731 cm^−1^, 1654 cm^−1^, 1497 cm^−1^, 1114 cm^−1^, 848 cm^−1^, and 750 cm^−1^ observed the same characteristic peaks at DNC PM as well. Although the peak of DNC/GA/PVP K30 SD has weakened, there was no shift of the characteristic peaks, indicating that the coordination interaction may not have taken place and DNC were not destructed during the process of ball milling [26,27]. The most likely reason for the weakening of characteristic peaks is that the DNC SD has less components of DNC. 

### 3.4. SEM

**Figure 5:**
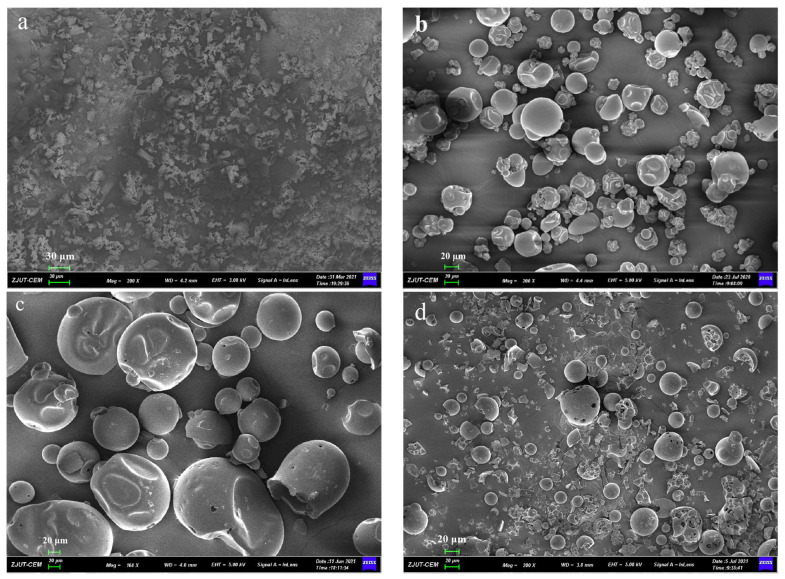

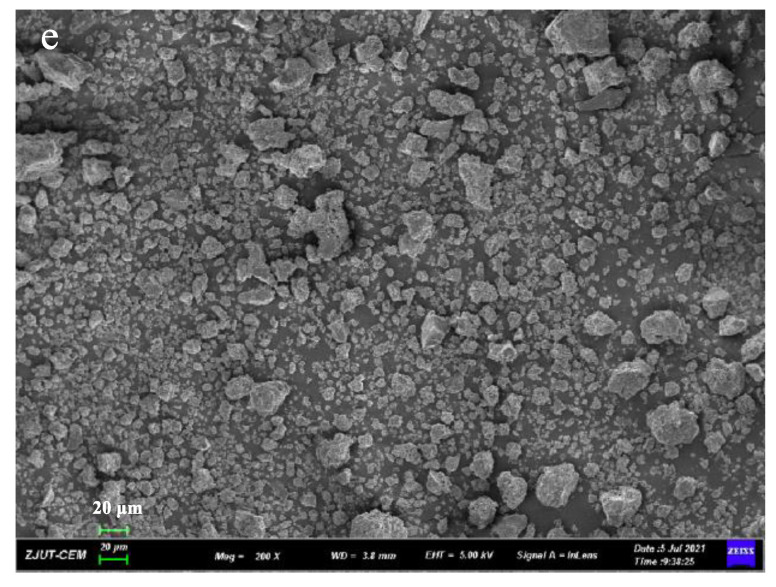
SEM images of (**a**) DNC, (**b**) GA, (**c**) PVP K30, (**d**) DNC/GA/PVP K30 PM, (**e**) DNC/GA/PVP K30 SD.

Microscopic surface morphology of all solid samples was shown in Figure 5. The pure GA (Figure 5b) and PVP K30 (Figure 5c) without processing have a spherical and porous surface structure. When GA, PVP K30, and DNC were milled in a planetary ball mill for 30 min, the original shape of spherical GA and PVP K30 was destroyed, and the formulation of DNC SDs had irregular smaller particles. The results indicated that mechanical ball milling could reduce the particles and have more irregular shapes.

### 3.5. Solubility

The water solubility results of DNC, DNC SDs, and corresponding physical mixture were shown in Table 2. The solubility of physical mixtures was slightly higher than that of DNC because of the solubilizing capacity of GA and PVP K30. The operation and equipment may cause the slight fluctuation of drug content, which was measured. In the binary system, the solubility of DNC was 451.33 μg/mL, which was 22,566 times as much as that of pure DNC; however, the ternary system (DNC/GA/PVP K30 SD, 921.44 μg/mL) showed a 46,072-fold increase. These results indicated that GA might self-assemble to form micelles in water to increase solubility, and become significantly outstanding when PVP K30 was added, which preliminarily confirmed that the possible combination of GA and PVP K30 plays a role in solubilization.

### 3.6. Particle Size, PDI, Zeta Potential, EE and LC

**Figure 6:**
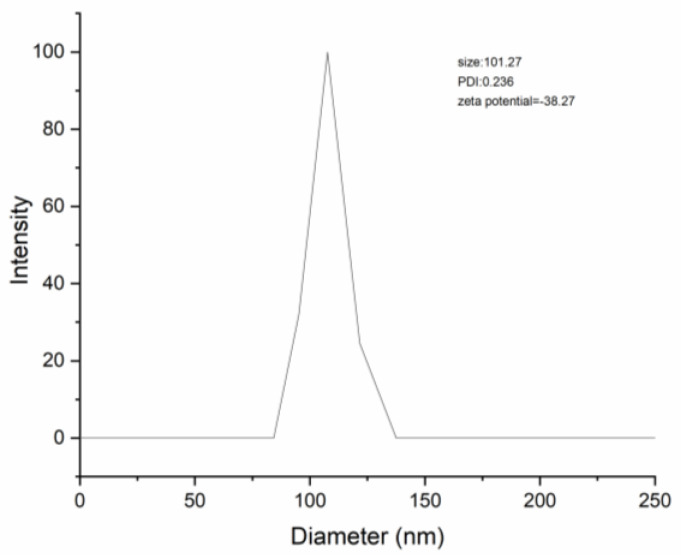
The particle size of DNC/GA/PVP K30 SD.

Table 3 presents the results of particle size, PDI, zeta potential, encapsulation efficiency, and loading capacity of DNC SDs. The particle size of DNC/GA and DNC/GA/PVP K30 micelles were 101.01 nm and 101.27 nm (Figure 6), while performing with the same dispersion index. Compared with the binary system, DNC/GA/PVP K30 micelles showed less zeta potential (−38.27), high EE (99.76%) and LC (18.13%). Therefore, it was manifested that encapsulation of DNC into GA polymeric micelles resulted in a homogenous and stable dosage form in aqueous solution with high drug loading. The advantage was more obvious by adding PVP K30 and being consistent with water solubility, which makes DNC water-soluble administration possible.

### 3.7. TEM

**Figure 7:**
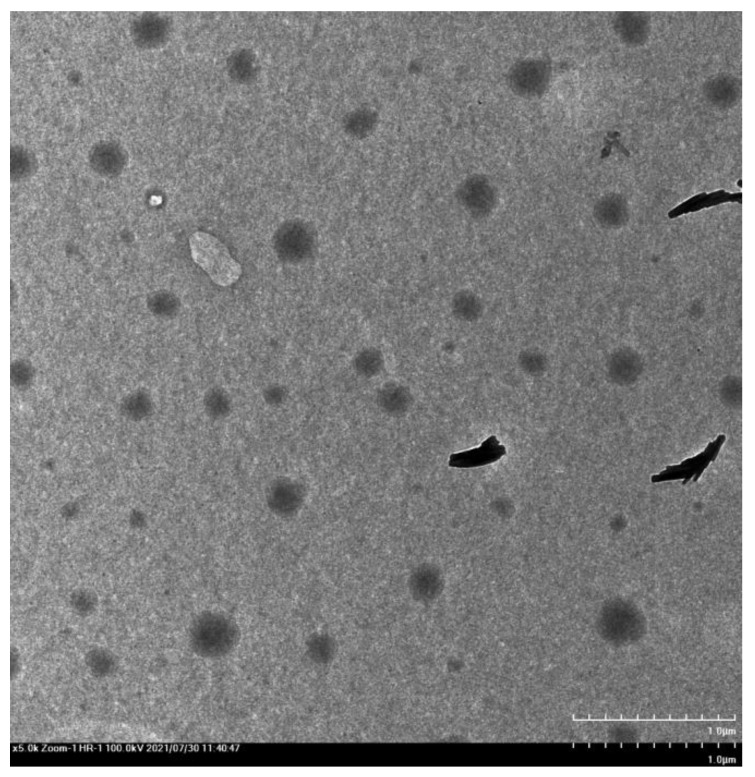
TEM image of DNC/GA/PVP K30 micelles.

The morphological characters of DNC micelles in water are as shown in Figure 7. DNC SD dissolves in water to form smooth spheroid micelles by self-assembly (Appendix A).

### 3.8. CMC

**Figure 8:**
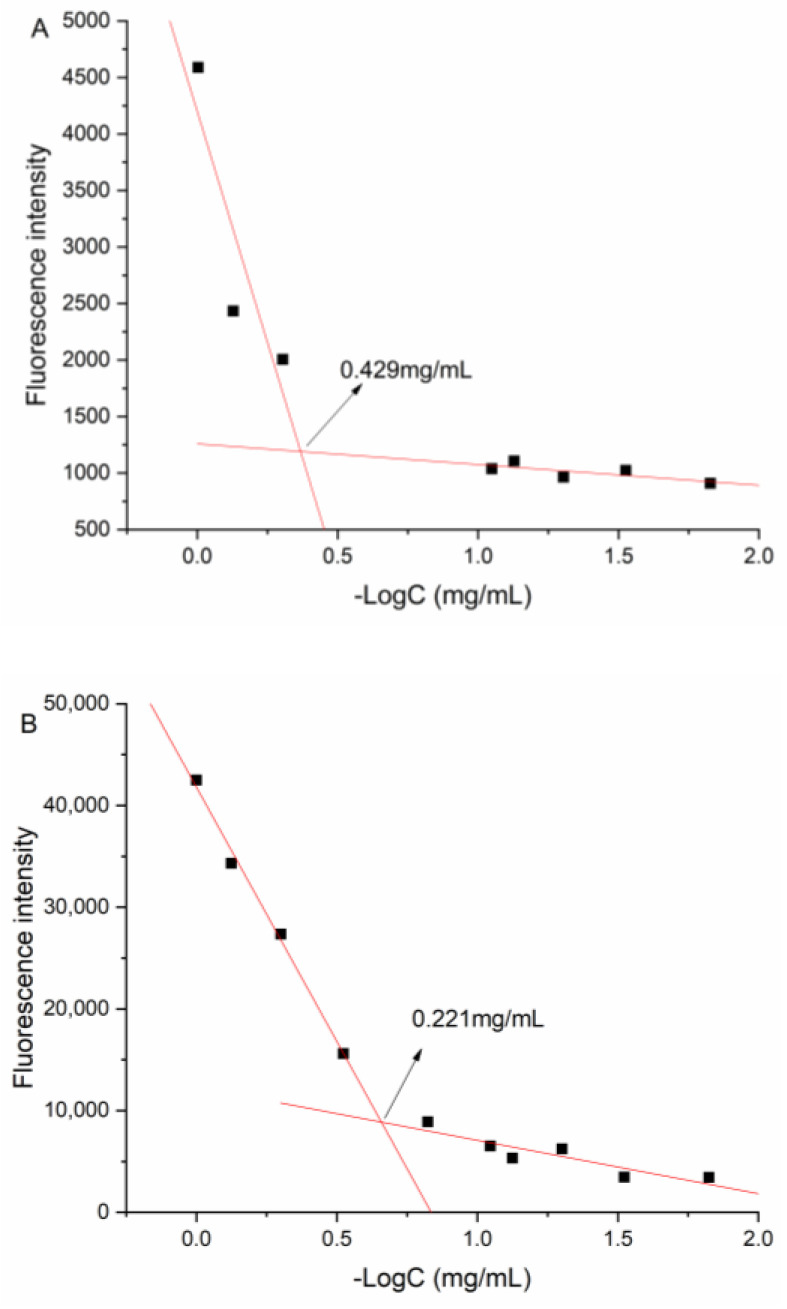
CMC value of (**A**) DNC/GA micelles; (**B**) DNC/GA/PVP K30 micelles.

The CMC value of DNC/GA micelles in an aqueous solution was 0.429 mg/mL, and the value of DNC/GA/PVP K30 micelles was 0.221 mg/mL, suggesting that the self-assembled micelles were extremely stable in the water solution (Figure 8). The increase of hydrophilicity could induce the enhancement of water solubility and decrease the CMC value further, which was consistent with the above results of water solubility. 

### 3.9. The Relaxation Time 

**Figure 9:**
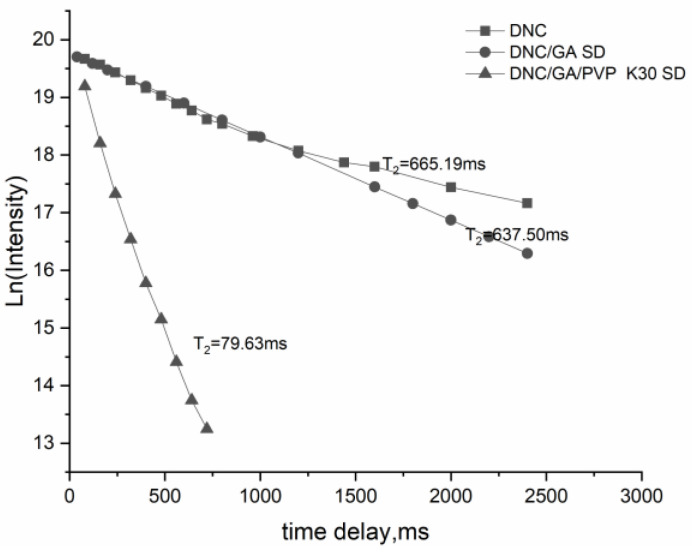
The relaxation time of DNC, DNC/GA SD, and DNC/GA/PVP K30 SD.

The method for the relaxation time of DNC and DNC solid dispersions by using an NMR relaxation study was discussed. The results of T_2_ relaxation times of the DNC binary and ternary system were 637.50 ms and 79.63 ms, Figure 9, shorter than DNC. Spin–spin relaxation times are related to molecular motions and binding state, the slow molecular mobility, and the closer the intermolecular interaction, the smaller the T_2_. It showed that the stabilization of DNC/GA/PVP K30 SD in D_2_O was clearly higher than that of the binary system, indicating that it accumulated in water to form more stable micelles [27]. 

### 3.10. In Vitro Drug Release

**Figure 10:**
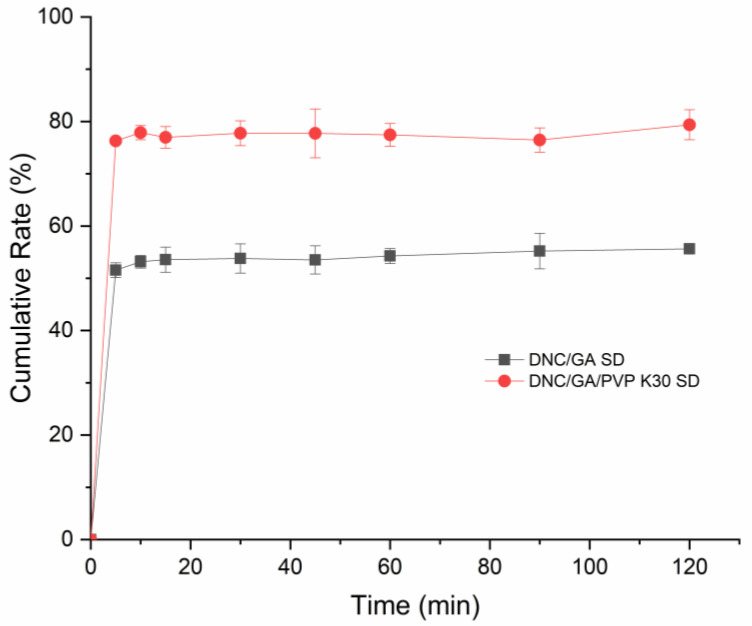
The cumulative release of DNC/GA SD and DNC/GA/PVP K30 SD.

The dissolution situation of DNS SDs was shown in Figure 10. In aqueous solution, the final release rates of the DNC/GA SD and DNC/GA/PVP K30 SD within 120 min were 55.6% and 79.38%. In general, the increase of solubility can improve the dissolution of drugs, while the amorphous drugs have a higher apparent solubility, which makes it easy to release drugs. For DNC/GA/PVP K30 SD, DNC was released very fast during the first five minutes and continued to increase its concentration up to 120 min. The pH of chicken intestine is almost 6.8 and digestion is fast, so it is desirable that the drug can be released rapidly in vivo and then exert drug efficacy. Experiment results revealed that DNC SDs could self-assemble into polymeric micelles in water and release in the intestine quickly.

### 3.11. Permeability

**Figure 11:**
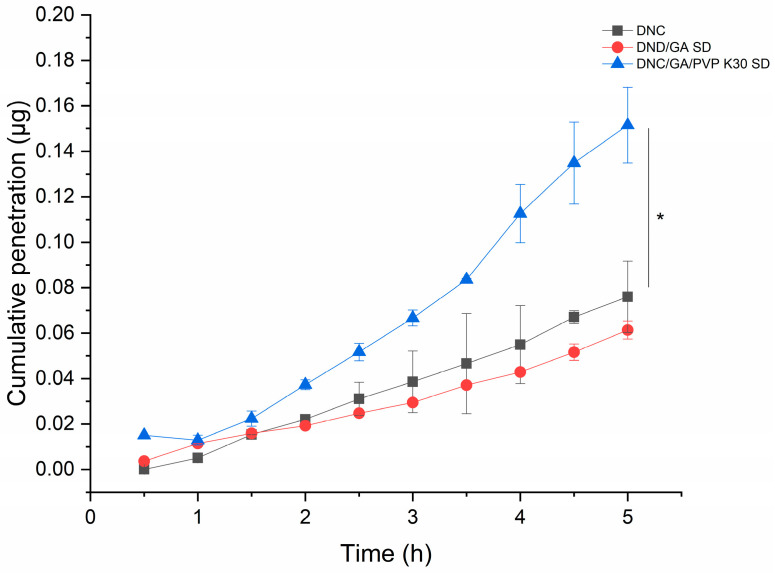
The cumulative penetration of DNC, DNC/GA SD, and DNC/GA/PVP K30 SD versus DNC, * *p* < 0.05.

The cumulative penetration of DNC/GA/PVP K30 SD was 0.152 μg and increased 2.5 times that of DNC, and the results were statistically significant, presented in Figure 11. Researchers conduct the PAMPA to predict the passive transmembrane ability of compounds. In this study, our finding indicated that the cooperation of GA and PVP K30 stimulated the passive transport of DNC in the intestine.

**Scheme 1:**
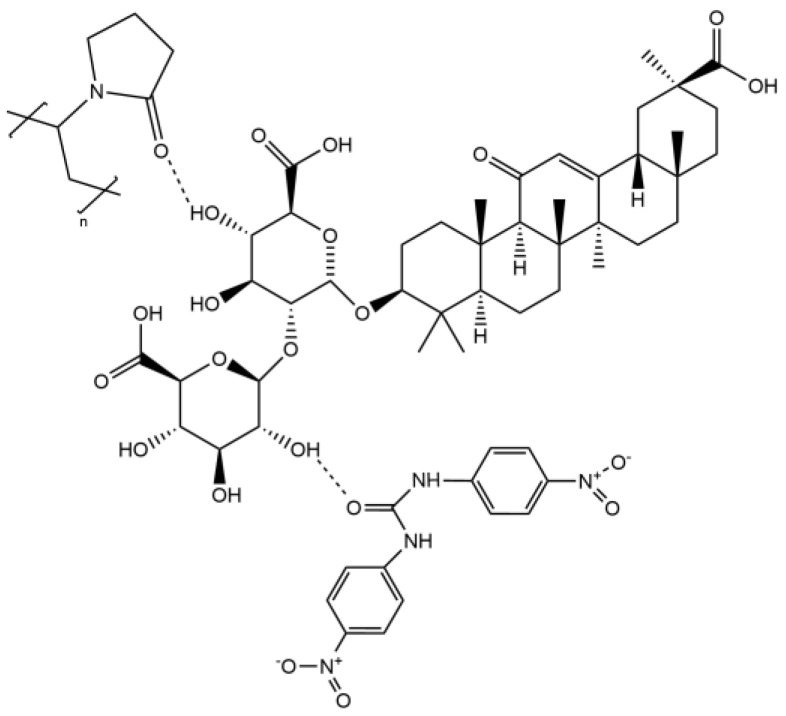
The hydrogen bonding of DNC, GA, and PVP K30 in water.

In the binary system, GA dramatically improved the solubility and release of DNC in aqueous solution by the mechanical process. When DNC/GA SD dissolved in water solution, the iminos of DNC and the carboxyl group of GA could form intermolecular hydrogen bonds to change the phenomenon of insoluble water. In order to select a better SD system, the DNC/GA/PVP K30 SD was prepared. The above results indicated that the ternary system surprised us more compared with the binary system, although it was also a dispersed amorphous state, and the water solubility and dissolution rate of it were significantly prominent. Our hypothesis on the DNC/GA/PVP K30 SD was as follows: firstly, there was not a chemical reaction during the mechanochemical ball milling process but only decreased the size to an amorphous state. Secondly, PVP K30 could interact with GA. The hydroxyl group of GA combined with the carboxyl group of PVP K30 to form hydrogen bonds when DNC/GA/PVP K30 SD was dissolved in water; the PVP K30 contained a hydrophilic end and the nitro group of DNC as a lipophilic group aggregated and self-assembled to form micelles (Scheme 1). Certainly, DNC may also have hydrogen bonding with PVP K30 or GA to produce DNC-GA and DNC-PVP micelles, and these micelles might present at the same time.

### 3.12. Pharmacokinetic 

**Figure 12:**
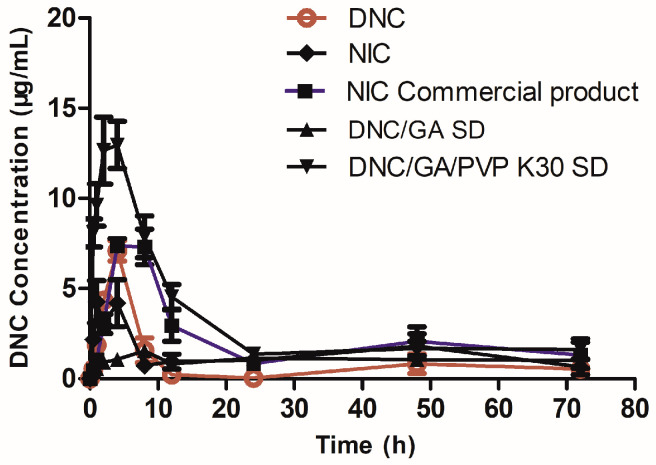
Concentration of DNC in plasma after oral administration.

As shown in Figure 12, profiles of plasma concentration time were determined after the oral administration of DNC and two species of DNC micelles in rats, respectively. After oral administration of DNC/GA/PVP K30 micelles, the T_max_ (time of maximum concentration), C_max_ (peak of plasma concentration), and AUC_0–72_ (area under the time curve) were 2.86 h, 14.46 μg/mL, and 238.35 μg/mL.h; however, the results of DNC were 4 h, 7.09 μg/mL, and 51.37 μg/mL.h, respectively (Table 4). The C_max_ and AUC_0–72_ in DNC/GA/PVP K30 micelles group were 2.04 times and 4.64 times higher than that for pure DNC (*p* < 0.01). It significantly proved that DNC/GA/PVP K30 SD self-assembled into micelles in water, enhanced the release rate, and improved the absorption of DNC in vivo by mechanical ball milling procession. Meanwhile, previous studies showed that the HDP possessed the property of facilitating the absorption [7]; in this research, the nicarbazin possessed higher oral bioavailability than DNC, which was consistent with it. Furthermore, the C_max_ of DNC/GA micelles was dramatically lower than pure DNC (*p* < 0.01), T_1/2_ was extended, and AUC_0–72_ was 309 μg/mL.h; it was speculated that DNC/GA micelles have a sustained-release effect and could maintain the blood concentration for a longer time to a certain extent, but the low drug concentration may have difficulty exerting the activity of DNC. The oral bioavailability of DNC/PVP K30 SD was increased, which may be down to certain factors. Firstly, the pharmaceutical dosage form changed, the DNC had strong hydrophobic compounds, and the solubility of DNC could significantly increase in water after being prepared DNC SDs by mechanochemical grinding to enhance the oral bioavailability. With the development of pharmacy, the emergence of new drug dosage forms, on the one hand, to ensure the long-term efficacy of drugs, and, on the other hand, greatly facilitates patients. The same dose lipophilic compounds revealed different oral bioavailability, and the solution is higher than suspension and powder is the worst. Secondly, DNC and the hydrophilic carrier material (GA and PVP K30) form a better micellar system in aqueous solution. The ternary system showed higher water solution, drug release, and permeation compared with a binary system, promoting the increase of blood concentration when DNC/GA/PVP K30 micelles enter into blood.

### 3.13. Tissue Distribution Study

**Figure 13:**
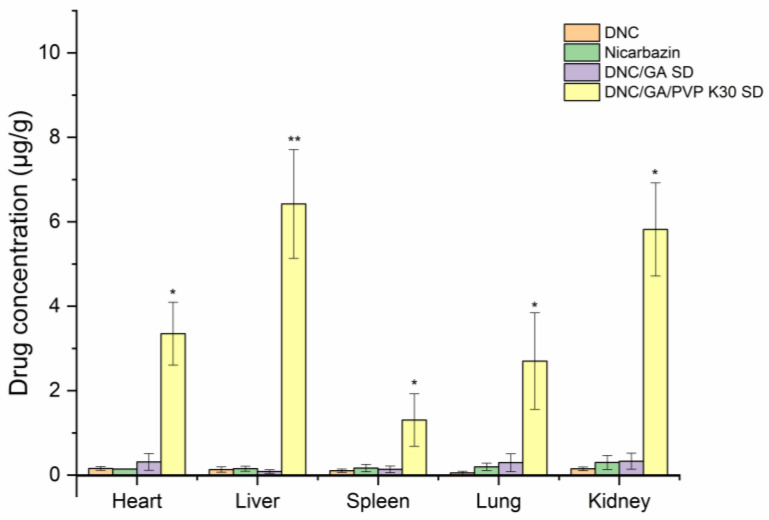
DNC concentration in tissue after oral administration (n = 6) versus DNC, * *p* < 0.05, ** *p* < 0.01.

Figure 13 presented the tissue drug distribution profiles of DNC after oral administration of pure DNC and two species of DNC micelles in rats, respectively. After continuous oral administration for one week, DNC was distributed into heart, liver, spleen, lungs, and kidneys, compared with that in pure DNC. The DNC concentration in DNC/GA/PVP K30 micelles group was significantly higher in these tissues. This study was consistent with bioavailability results; when the drug entered into the bloodstream, it was absorbed by the liver and distributed throughout the body, finally excreted in the kidneys. 

The figure showed that the accumulation of DNC in the liver tissue was 6.42 μg/mL, which was the largest. Warren [28] found that nicarbazin had inhibited the activity of hepatosplenic shistosomiasis mansoni in mice and there was an improvement in sustainability after terminal treatment. Antunes et al. [29] reported the suppressive activity on oviposition in schistosoma mansoni infection. Due to the liver-targeting aggregation effect of DNC/GA/PVP K30 micelles, it could hypothesize that the DNC ternary system may have a strong inhibitory effect for hepatosplenic shistosomiasis mansoni and markedly ameliorates early disease. At the beginning of the experiment, the NIC commercial product was administrated as well; however, the study animals had unexplained deaths, and all of them died before the experiment was over. The results showed that the NIC commercial product was toxic to rats, which indicated a safety hazard for human beings and the importance of new product development.

### 3.14. Assessment of Anticoccidial Activity

#### 3.14.1. Clinical Symptoms

**Figure 14:**
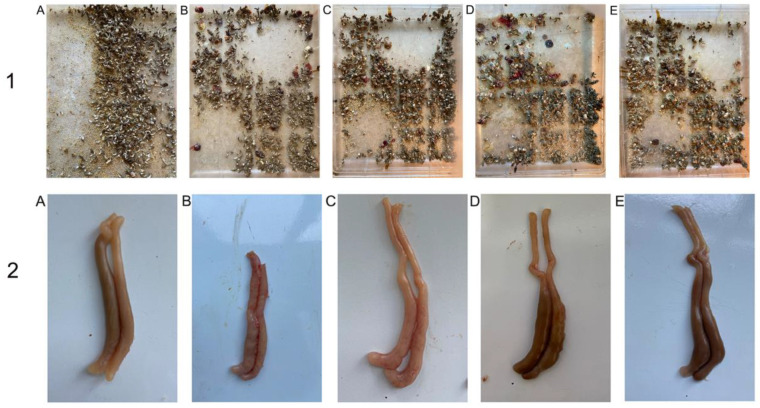
The images of hematochezia (1) and cecum (2) of chicken. (**A**) NC, (**B**) PC, (**C**) DNC, (**D**) NIC commercial product, (**E**) DNC/GA/PVP K30 SD.

After being infected with *E. tenella*, the PC showed disheveled feathers, dullness, and anorexia. These symptoms in DNC, NIC commercial product, and DNC/GA/PVP K30 groups were much milder. From the 5th day on, the appearance of hemorrhaging in feces were noted except NS; the 6th day was most serious, indicating that the model was successfully established. The DNC/GA/PVP K30 group had the least bloody stools, suggested that DNC SD could alleviate the appearance of hemorrhages, as showed in Figure 14-1. There was no dead chick throughout the experiment, thus the mortality rate was 0% in each group.

#### 3.14.2. Lesion Scores

Figure 14-2 presented the pathological lesion scores of study groups. PC observed severe pathologic lesions, which included atrophy, wall thickening, erosion, and dark blood clotting. The ceca in DNC and NIC commercial products displayed various degrees of morphology. According to pathologic performance, the cecum was scored, and the more severe, the higher the grades. It was clear that the lesion scores of NC and PC were 0 and 28, and DNC/GA/PVP K30 obtained a lower score than PC. Results suggested that both cecal lesions symptoms were relieved with DNC/GA/PVP K30. 

#### 3.14.3. Relative Weight Gain Rate

The average gain weight of chicks in PC and DNC were 44.8 g and 47.2 g, respectively, and relative weight gain rates were 82.05% and 86.45%, as shown in Table 5. After being infected with coccidiosis, the PC showed a decrease in food intake induced decreased weight gain, with a lower RWG than the other groups; although DNC could alleviate this to some extent, the effect was minimal without HDP. After prophylactic administration with DNC/GA/PVP K30, the RWG up to 96.66% was better than the NIC commercial product.

#### 3.14.4. Oocysts per Gram Output and ACI

Except for NC, the oocysts in feces could be detected, and the number of oocysts significantly decreased after the administration of DNC/GA/PVP K30. The relative inhibition rate of oocysts of DNC, NIC commercial product, and DNC/GA/PVP K30 SD were 49.01%, 34.95%, and 77.36%, as shown in Table 6. The PC showed a non-anticoccidial effect, and the ACI index was 114.05. ACI indices of DNC and NIC commercial product groups were 127.45 and 135.67 (Table 7), which were regarded as inefficient anticoccidial agents. The DNC SD treated group showed moderate anticoccidial effects, and the ACI index was 167.67, Table 7.

When chickens eat infective sporozoites, it reaches the cecum in the digestive tract and infects cecal epithelial cells into capillaries, with blood in the intestinal gland to complete the fission and sexual reproduction stage, and then penetrating the intestinal villous epithelial cells into the intestinal lumen to destruct the cecum with some symptoms occurring such as hemorrhaging in feces, disheveled feathers, dullness, anorexia, and inducing mass fatality in severe cases [30]. In order to reduce the risk of infection, farms usually for sustainability overused anticoccidial drugs. Due to adding them long-term to the diet, the oocysts used in the experiment may have developed some resistance to nicarbazin active ingredients and may be the reason for the low ACI value of the experimental NIC commercial product. Although HDP was an inactive substance and enhanced the activity of DNC 10-fold, researchers presumed, by increasing absorption, that the water solubility has not been improved, which was still inconvenient for drug administration. In this research, we used DNC (NIC active compound), GA, and PVP K30 to prepare solid dispersion without any organic solution, not only enhancing water solubility and oral bioavailability, but also the anticoccidioidal capability being better than NIC commercial products. Moreover, there was a relationship wherein the ACI increased with an enhancement in bioavailability, and the increased order as follows: DNC, NIC commercial product, and DNC/GA/PVP K30. Based on previous results, HDP enhanced 10-fold the anticoccidioidal capability of DNC by facilitating absorption, and we speculated that the DNC ternary solid dispersion system prepared by mechanochemistry could enhance the efficacy in the same certain, exceeding that of the co-crystal compound nicarbazine composed of HDP and DNC.

### 3.15. Stability Study

**Figure 15:**
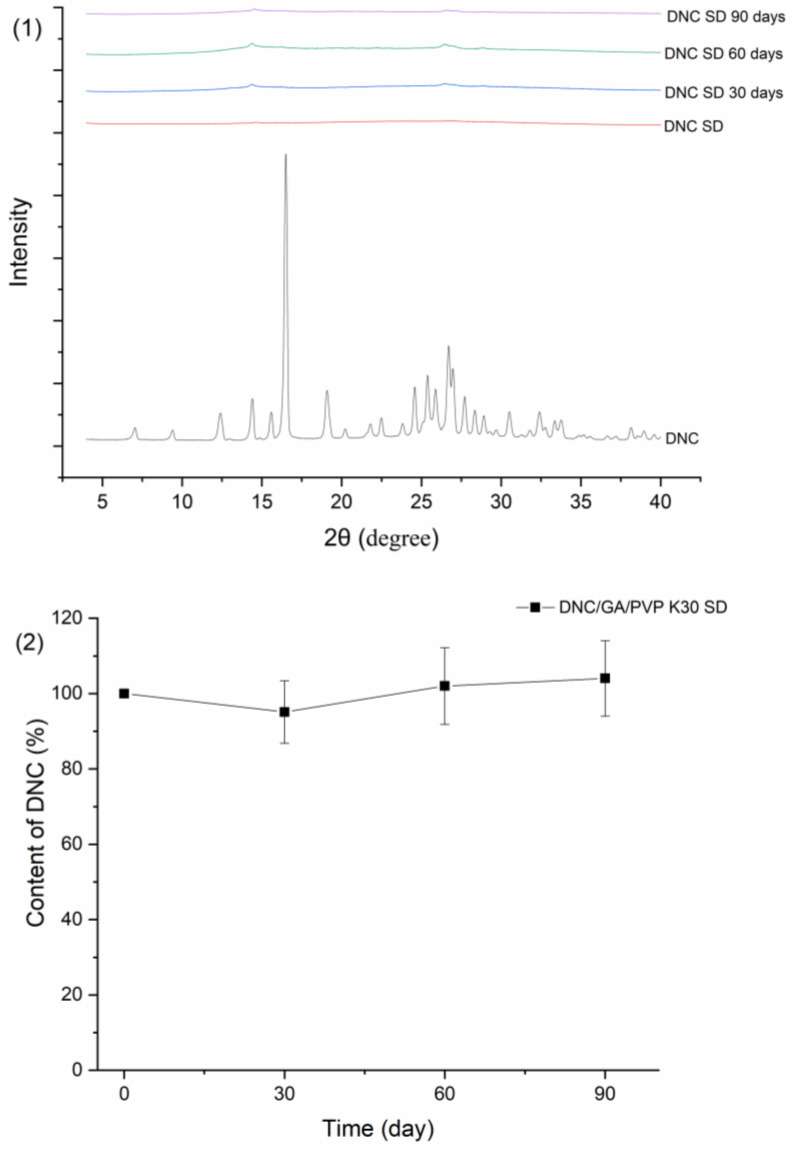

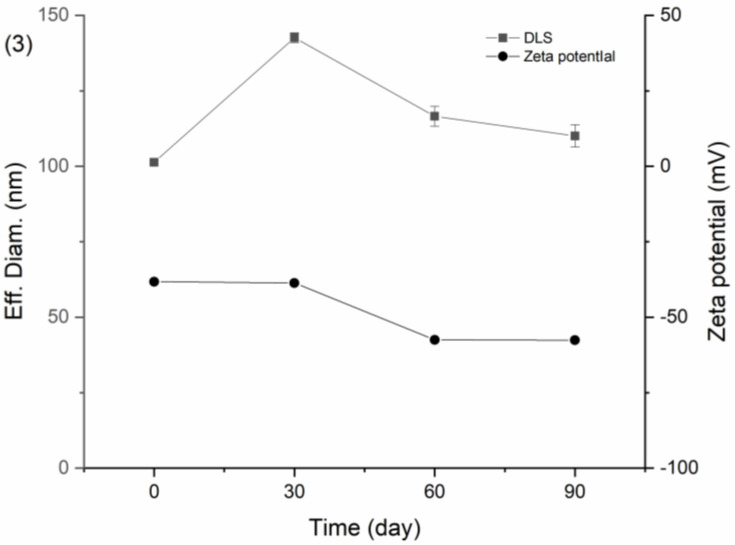
Stability test of (**1**) PXRD, (**2**) Drug content, (**3**) Particle size, and zeta potential of DNC/GA/PVP K30 SD.

Figure 15 showed the results of DNC/GA/PVP K30 SD of PXRD, drug content, particle size, and zeta potential after three months of storage. As shown below, DNC/GA/PVP K30 SD has no significant crystal transformation and is still in a relatively amorphous state. Meanwhile, the DNC content of SDs, particle size, and zeta potential remained relatively steady compared with the original state. For DNC/GA/PVP K30 SD, which was packed with an aluminum foil bag, the size of particles increased after 30 days conjecturing that the package of that batch was not sealed completely, causing moisture absorption and agglomeration, and then decreased, indicating that DNC solid dispersion could keep the drug stable for a certain time. This evidence manifested that DNC SDs that were prepared by a mechanochemical force could reduce the generation of outgrowth, maintaining stability during the storage process.

## 4. Conclusions

In this investigation, DNC/GA/PVP K30 solid dispersion was successfully prepared via mechanical ball milling, which self-assembled to form DNC micelles in water and assigned the therapy of coccidiosis in vivo. The characterization of solid state results demonstrated that DNC was dispersed in GA/PVP K30, the crystal disappeared into an amorphous state, there was no chemical reaction during the ball milling process, and stability can be maintained in a certain period. When DNC/GA/PVP K30 SD dissolved in aqueous solution, the solubility of DNC enhanced 46,072 times that of pure DNC, and the drug release behavior in vitro was significantly improved. After the DNC was entrapped to form micelles, the concentration of DNC in blood and tissue enhanced, and the anticoccidial effect was correspondingly increased compared with free drugs, which makes it possible for animals to be administrated DNC drug through drinking water. In addition, the accumulation of DNC micelles in the liver tissue may have potential therapeutic and preventive effects on schistosomiasis. Thus, the research elucidated an innovative preparation of an amorphous DNC SD formulation by mechanical ball milling and might be a promising formulation to increase the therapeutic.

## Figures and Tables

**Table 1:** Conversion between oocysts percent gram and oocysts value.

OPG/PC OPG	<1%	1–25%	25–50%	50–75%	75–100%
Oocysts value	0	5	10	20	40

OPG/PC OPG = 100*Oocysts/g output of per group/Oocysts/g output of the PC group.

**Table 2:** The water solubility of DNC SDs and DNC PMs.

Sample	Solubility (μg/mL)	Increasing Times	Content (%)
DNC	0.02	-	100
DNC/GA SD	451.33	22,566.50	100.91
DNC/GA PM	0.10	5.00	105.18
DNC/GA/PVP K30 SD	921.44	46,072.00	96.89
DNC/GA/PVP K30 PM	1.55	77.50	105.46

**Table 3:** The particle size, PDI, zeta potential, EE, and LC of DNC SDs.

Sample	DNC/GA SD	DNC/GA/PVP K30 SD
Size (nm)	101.01 ± 0.07	101.27 ± 0.64
PDI	0.24 ± 0.01	0.236 ± 0.01
Zeta potential (mW)	−31.15 ± 0.68	−38.27 ± 0.21
EE (%)	69.39%	99.76%
LC (%)	13.87%	18.13%

**Table 4:** Pharmacokinetic parameters of DNC by oral administration (n = 6).

Parameter	DNC	NIC	NIC CP	DNC/GA SD	DNC/GA/PVP K30 SD
*C*_max_ (μg/mL)	7.09 ± 0.97	5.99 ± 2.13	8.29 ± 0.82 ^*^	1.89 ± 0.58 ^**^	14.46 ± 1.41 ^**^
*T*_max_ (h)	4.00 ± 0.00	4.00 ± 0.00	5.2 ± 2.68	7.20 ± 1.79 ^**^	2.86 ± 0.99 ^*^
*T*_1/2_ (h)	25.28 ± 4.01	22.91 ± 4.54	17.68 ± 6.51	95.60 ± 31.06	24.55 ± 3.85
AUC_0__→72_ (μg/mL.h)	46.49 ± 11.83	79.82 ± 23.35 ^*^	107.32 ± 28.45 ^**^	93.78 ± 13.67 ^*^	238.35 ± 27.73 ^**^
AUC_0__→__∞_ (μg/mL.h)	67.11 ± 20.83	87.14 ± 27.06 ^*^	116.018 ± 34.61 ^**^	239.30 ± 114.05 ^*^	287.03 ± 72.75 ^**^

Compared with DNC, ^*^ *p* < 0.05, ^**^ *p* < 0.01.

**Table 5:** The relative weight gain rate of each group.

Sample	NC	PC	DNC	NIC CP	DNC/GA/PVP K30 SD
The average weight gain (g)	54.60 ± 8.04	44.80 ± 16.18	47.20 ± 6.28	50.00 ± 6.14	52.78 ± 6.14
Relative weight gain rate (%)	100	82.05	86.45	91.57	96.66

**Table 6:** The oocysts percent gram of each group.

Sample	NC	PC	DNC	NIC CP	DNC/GA/PVP K30 SD
Oocysts percent gram/(×10^6^)	0 ± 0	30.33 ± 1.16	15.47 ± 0.66	19.76 ± 1.11	6.88 ± 0.74
Relative inhibition rate (%)	-	0	49.01	34.95	77.36
Oocysts value	0	40	20	20	5

**Table 7:** ACI.

Sample	NC	PC	DNC	NIC CP	DNC/GA/PVP K30 SD
Survival rate (%)	100	100	100	100	100
RWG rate (%)	100	82.05	86.45	91.57	96.67
Cecum score	0	28	19	16	9
Oocysts value	0	40	20	20	5
ACI	200	114.05	147.45	155.57	182.67

**Figure A1:**
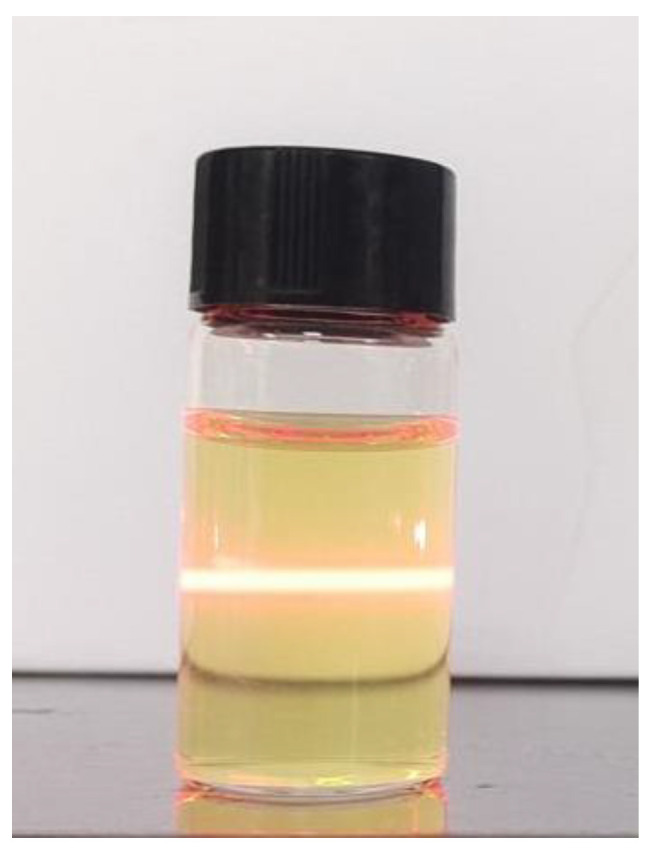
DNC/GA/PVP K30 SD was dissolved in water solution.

## Data Availability

Not applicable.

## Appendix A

When DNC/GA/PVP K30 SD was dissolved in water, the Tyndall effect appeared, indicating that solid dispersion was evenly dispersed in water to form micelles.
